# Telemedicine as a “digital umbilical” for maternal and reproductive healthcare: a qualitative study from health and wellness centers in rural West Bengal, India

**DOI:** 10.1186/s12913-026-14916-1

**Published:** 2026-07-06

**Authors:** Sibasis Mandal, Preeti Dhillon, Jayanta Das

**Affiliations:** 1 Department of Survey Research & Data Analytics, International Institute for Population Sciences, Mumbai, India; 2 Department of Public Health and Mortality Studies, International Institute for Population Sciences, Mumbai, India

**Keywords:** Telemedicine, Hybrid care, Community health officer, Maternal health, Reproductive health, Health and wellness centre, Rural health services, India, Digital health, Qualitative research

## Abstract

**Background:**

Rural women in India continue to face major geographical, financial, and sociocultural barriers to get timely maternal and reproductive healthcare, despite recent investments in primary care infrastructure and digital health initiatives. The West Bengal state telemedicine program, where patients are delivered healthcare through Health and Wellness Centres (HWCs), offers a hybrid model that combines on-site Community Health Officers (CHOs) with remote physicians, but its functionality for reproductive health remains underexplored.

**Methods:**

This descriptive qualitative study explored staff experiences of implementing and delivering a hybrid telemedicine model for maternal and reproductive healthcare at peripheral Health and Wellness Centers (HWCs) in two West Bengal districts selected for their differing health system capacities. In total, 23 in-depth interviews were conducted with CHOs, Medical Officers, IT staff, and program administrators involved in telemedicine systems at HWCs and linked facilities in the selected two districts (Nadia and Howrah). The collected interview data were analyzed using reflexive thematic and sentiment analysis.

**Results:**

Participants described telemedicine as a bridge that connects rural women, especially older, low-income, and socially disadvantaged clients for getting specialist guidance while preserving free medications, reducing travel, and strengthening continuity of care for general and reproductive health needs. Across the reproductive continuum, teleconsultations supported safer family planning, triage and co‑management of high‑risk pregnancies, and follow‑up for minor post‑partum complications, anchored by CHOs’ physical assessments. In the studied telemedicine system, recurrent barriers include doctor unavailability, pressure to meet daily case targets, incomplete clinical information, and weak digital infrastructure. These led to rushed consultations, generalized prescriptions, and limited suitability of telemedicine for emergencies or complex gynecological assessment.

**Conclusions:**

Hybrid telemedicine at HWCs can substantially narrow access gaps in maternal and reproductive healthcare in rural settings when CHOs are empowered as skilled on-site assessors and consistent remote clinical support is available. To realize its full potential, programs must rebalance quantity-driven targets with quality, invest in joint training for CHOs and physicians, and address persistent infrastructural bottlenecks so that telemedicine can function as a reliable screening and support system rather than a substitute for essential in‑person care.

**Supplementary Information:**

The online version contains supplementary material available at https://doi.org/10.1186/s12913-026-14916-1.

## Background

Ensuring everyone has access to good maternal and reproductive healthcare is still a major, ongoing public health challenge, particularly across low and middle-income regions [1, 2]. People living in remote rural areas experience significantly worse health outcomes than their urban counterparts, with distance acting as a major economic and social barrier that often prevents vulnerable populations—particularly women—from accessing the specialised medical care they need, resulting in higher risks of maternal mortality and severe morbidity due to delays caused by transport costs and gendered power relations [3–7]. In India, maternal mortality has declined over the past decade, yet substantial inequalities in the utilisation of maternal health services persist, with rural, poorer, and socially marginalised women less likely to receive adequate antenatal, delivery and postnatal care [8]. Traditional, capital-intensive strategies like building new hospitals and extensive specialist training are slow and often fail to meet the pace of population growth and increasingly complex epidemiological needs [9–12].

Over the past several decades, digitally mediated capacities have revolutionised progress in individual health and medical management, as well as population-wide health strategies that promote digital health [13, 14]. Nowadays, digital health interventions have transitioned from experimental or supplementary roles to systemic necessities. Specifically, telemedicine is now strategically operationalised as a central and critical component of primary healthcare delivery, not merely an add-on service [15–18]. Telemedicine, in particular, serves its most vital function by bridging the chronic shortage of Medical Officers (MOs) and specialists (e.g., gynaecologists and obstetricians) in rural areas, where establishing and staffing full‑scale specialist services is both slow and expensive [19, 20]. Simply establishing a digital connection is insufficient, as direct-to-consumer digital health often fails to reach the most vulnerable populations: the poor, those with limited digital literacy, and the elderly, due to their lack of access to technology or foundational digital skills [21, 22]. This highlights the need for models embedded in primary care facilities.

Population ageing is widely recognised as a structural driver of rising health expenditure, as older populations require more intensive management of chronic and long‑term conditions, raising concerns about the fiscal sustainability of health systems [23, 24]. Evidence from health economics and policy analysis highlights digital health and telemedicine as strategies to moderate cost growth by improving efficiency and shifting some care from high‑cost hospital settings to lower‑cost remote encounters [24]. The WHO’s second global survey on eHealth underscores how telemedicine can reduce travel, waiting time, and indirect costs for patients while optimising use of scarce specialist resources, particularly in rural and underserved areas [25]. Empirical evidence from the U.S. Veterans Affairs system shows that telemedicine visits saved patients on average about 145 miles and 142 min of travel per visit, alongside measurable reductions in travel reimbursement expenditures, illustrating the concrete cost and time savings that such models can generate [26].

India is a notable example of how telemedicine can be successfully and systematically integrated into a large public health system, largely by upgrading the existing infrastructure [27, 28]. The modern digital healthcare system is built on a national network of Health and Wellness Centres (HWCs), these redesigned Sub-Centres bring Comprehensive Primary Health Care right to the communities close to them. HWCs are essentially the physical hubs for a massive digital health network, and telemedicine is a key component, it’s become a non-negotiable part of their operations. This whole system is intentionally set up to tackle the significant “resource gap”, particularly the serious shortage of doctors and specialists in rural and smaller urban areas [29–31]. India has invested heavily in a national network of Health and Wellness Centres (HWCs), which upgrade former sub‑centres to deliver comprehensive primary healthcare close to communities. HWCs function as physical hubs within a wider digital health ecosystem, with telemedicine now a routine, non‑negotiable component of their operations. In West Bengal, the “Swasthya Ingit” telemedicine programme connects HWCs to hub facilities (Primary Health Centres, rural or district hospitals, and tertiary centres) through a spoke‑and‑hub model: CHOs at HWCs act as the “spokes” providing on‑site assessment, basic investigations, and counselling, while remote MOs and specialists at higher‑level facilities act as “hubs” offering diagnostic and treatment guidance via audio or video consultations.

The ecosystem is designed and operates on a highly efficient spoke-and-hub model [32] of resource distribution and service delivery. In this structure:

- The Spoke: The Community Health Officer (CHO), stationed at the HWC, serves as the *spoke*. They are the crucial grassroots workers, responsible for delivering primary services, conducting physical assessments, utilising diagnostic tools, and providing crucial on-site support.
- The Hub: The Medical Officers or Specialists, located at PHCs, Rural Hospitals, district hospitals or tertiary care centres, function as the *hub*. They provide remote, expert consultation, diagnosis, and treatment guidance.

This blended telemedicine model is rooted in a physical site, such as Health and Wellness Centres, and operates through a tripartite system involving patients, CHOs as on-site skilled assessors, and remote Medical Officers (MOs) or physicians delivering specialist oversight. CHOs serve as the essential physical bridge for assessments and patient engagement, while MOs provide remote expertise, supported by dedicated personnel handling digital systems, scheduling, and data coordination [33]. This hybrid framework combines hands-on evaluation with remote efficiency, as evidenced in India’s ‘eSanjeevani’ and West Bengal’s ‘Swasthya Ingit’ initiatives.

**Fig. 1:**
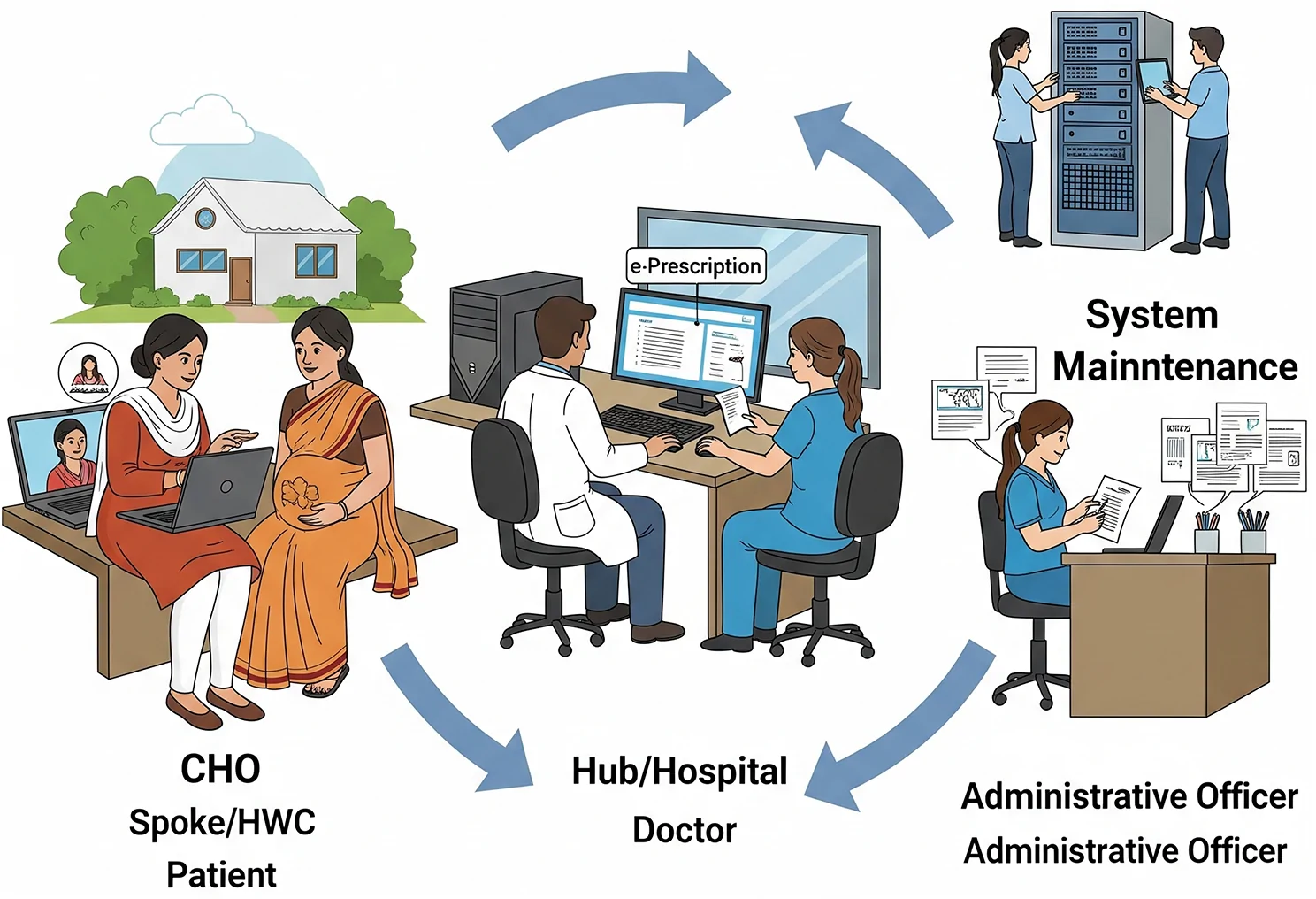
Practised telemedicine model in rural India at the government health facility

This approach is inherently anchored in a physical space and relies on a robust three stakeholder operational system. This integrated approach, involving the patient, the CHO as the skilled on-site assessor, and the remote physician providing expert guidance, is vital. It combines necessary physical assessment and patient interaction with the efficiency of remote specialist expertise. CHOs are the indispensable physical interface, MOs offer critical remote expertise, and dedicated staff manage the digital infrastructure, scheduling, and data flow [32, 34]. In practice, telemedicine consultations for maternal and reproductive health typically follow a standard workflow. During routine service hours at HWCs, CHOs register women, take histories and vital signs, and enter clinical information into the state portal before initiating a call to a hub doctor. Audio‑video calls allow women to speak directly with the doctor while the CHO presents examination findings; prescriptions are printed at the HWC and medications dispensed from on‑site stocks. Teleconsultations are used across the reproductive continuum—for family planning (e.g. injectable contraceptives), antenatal risk assessments, referral of high‑risk pregnancies, and follow‑up of minor postpartum complications—while women still attend HWCs or higher‑level facilities in person for examinations, laboratory tests, and delivery care. The practiced model is shown in Fig. 1.

The HWC telemedicine system instantly and significantly expands patient access to qualified physicians for continuous management of prevalent non-communicable diseases (hypertension, diabetes) and minor ailments (fever, cough, gastritis). Crucially, it provides vital access to specialised consultations that are often unavailable in rural areas, including oncology, neurology, mental health, cardiology, and gynaecology [31, 33, 35–37]. In the crucial domain of reproductive health, telemedicine is actively employed across the entire care continuum, from preventive services to complex management [38–40]. This includes:

- Facilitating prescription approval and counselling for numerous family planning methods.
- Aiding in the management of high-risk antenatal complications, such as severe anaemia (low haemoglobin levels) or gestational hypertension (high blood pressure) and provide assistance (e.g. clinical assessment support, prescription and medicine dispense and explain those to patients, and follow‑up counselling etc.).
- Supporting essential postpartum care, including counselling and monitoring and treatment of unwanted complications.

Considering the inherent and recognised limitations of remote assessment (e.g., the inability to perform physical examinations that require direct contact), the system is optimally designed to function as an efficient screening, triage, support, and education tool, enabling the identification of high‑risk cases for timely referral. Within this national architecture, West Bengal has developed its own state telemedicine programme, which uses Health and Wellness Centres in rural districts to deliver hybrid teleconsultations that aim to ease travel, time, and out‑of‑pocket burdens for rural users, including women seeking maternal and reproductive care in remote villages. A comprehensive in-depth understanding is urgently required concerning the specific operational scope, detailed mechanisms of function, validated actual successes, and ongoing challenges in the practical application of telemedicine to these essential and life-saving maternal and reproductive health services.

Given the rapid expansion of telemedicine in India, there is limited qualitative evidence on how hybrid, CHO‑assisted teleconsultations function specifically for maternal and reproductive healthcare within public Health and Wellness Centres (HWCs), particularly in eastern states like West Bengal. Most prior studies focus on general telemedicine use or non‑reproductive conditions, with little attention to the sociological and clinical implications of routing reproductive care through digital platforms anchored in primary care. This study therefore aimed to explore the operational, sociological, and clinical dimensions of implementing this hybrid model across the full reproductive health continuum—from family planning to postpartum care. Drawing on experiences of frontline CHOs, consulting Medical Officers (MOs), and administrative staff, we sought to understand practical implementation, clinical usefulness, and key challenges, offering nuanced insights into how telemedicine can function as a “digital umbilical” for rural women’s reproductive care and where it currently lacking.

## Materials and methods

### Study design and setting

We conducted a descriptive qualitative study to explore staff experiences with implementing and delivering the “Swasthya Ingit” hybrid telemedicine model for maternal and reproductive healthcare at peripheral Health and Wellness Centres (HWCs) in West Bengal. Although the service caters to a broad range of health conditions, this study focused specifically on its application across the reproductive health continuum, including family planning, antenatal care, intrapartum‑related referrals, and postpartum care. Using reflexive thematic analysis, we examined how CHOs, Medical Officers (MOs), IT staff, and administrative doctors perceived the implementation process, clinical utility, and challenges of this hybrid model across HWCs and their linked facilities. The research was conducted in two West Bengal districts, Howrah and Nadia, selected based on a Ministry of Health and Family Welfare press release to represent districts with fair and weak health infrastructure, respectively [41].

### Characteristics of the participants and sampling

We used a multistage sampling strategy. First, using Ministry of Health and Family Welfare classifications of district health system capacity, we compiled a list of eligible districts and then randomly selected two: Howrah (classified as having ‘fair’ health infrastructure) and Nadia (classified as ‘weak’), allowing us to examine telemedicine implementation under contrasting infrastructural conditions. Second, within each district, we systematically selected two rural blocks—one located closer to the district headquarters and one more remote—where the ‘Swasthya Ingit’ telemedicine programme was well established. Third, within these blocks, we purposively recruited staff directly involved in telemedicine operations (CHOs, Medical Officers, IT staff, and administrative officers) to capture diverse operational and clinical perspectives. In total, we enrolled 23 participants: 15 CHOs from rural Health and Wellness Centres, 6 physicians/medical officers from primary, rural, and higher-level facilities, and 2 district-level IT personnel (Table S1). According to district records, approximately 977 CHOs and 136 eligible MOs, administrative officers, and IT staff were available across the two districts; recruitment aimed to capture variation in cadre, facility level, and context, and continued until thematic saturation, defined as the point at which no substantively new codes or themes emerged from successive interviews.

### Inclusion criteria

The study included CHOs, doctors, IT staff, and administrative officers who were actively engaged in state telemedicine services within the two selected districts of West Bengal. Only individuals who were willing to participate and provided informed consent were included.

### Data collection tool and technique

To guide the in-depth interviews (IDIs), we developed a semi-structured interview guide based on a review of telemedicine and maternal health literature and consultation with senior qualitative researchers and programme experts. The guide was piloted with CHOs in two HWCs in Nadia district, and minor refinements were made to question order and wording to improve clarity and flow. The final guide covered domains such as telemedicine workflow, experiences with maternal and reproductive health consultations, perceived benefits and challenges, and suggestions for improvement; the guide is provided as Table S2.

A total of 23 face-to-face IDIs were conducted in Bengali, the local language, to facilitate effective communication. Before each interview, the interviewer explained the study purpose, procedures, risks and benefits, and confidentiality safeguards; written informed consent was then obtained for both participation and audio-recording. The first author conducted and audio-recorded all interviews, taking care not to record or minimise any information that could reveal a respondent’s personal identity; transcripts were de-identified by removing names and other direct identifiers, and pseudonyms were used in all quotations. Only de-identified transcripts were analysed. The interviews analysed in this paper took place between May and October 2025, when the Swasthya Ingit telemedicine programme was fully operational in both districts and staff availability allowed in-depth interviews alongside routine service delivery, within a broader fieldwork period during which we also collected data on other telemedicine interactions beyond the maternal and reproductive domain. For participants’ convenience, interviews were conducted at their respective workplaces, including HWCs, Primary Health Centres (PHCs), hospitals, and administrative offices.

The audio recordings were transcribed verbatim into Bengali and then translated into English. The English translations were carefully reviewed and corrected for clarity and grammar before being entered into a Microsoft Word document, and data familiarisation was achieved through repeated reading of the transcripts and initial note-taking.

### Ethical statement

The study received ethical approval from the Student Research Ethics Committee of the International Institute for Population Sciences (SERC no.: IIPS/AC/SREC/SM/IO-312/2025) and permission from the West Bengal State Health Department (Memo no.: HFW-18099/17/2025-PHP SEC-Dept. of H&FW/3(8)). Written informed consent was obtained from all participants after explaining the study purpose and their right to decline participation or withdraw at any stage. To ensure confidentiality and anonymity, pseudonyms were used, participation was unrelated to employment appraisals, and interviews were scheduled to minimise workplace pressure, consistent with ICMR guidance on avoiding undue influence. All data are stored securely for five years, accessible only to the research team, after which they will be permanently deleted; the study adhered to the Declaration of Helsinki, the Indian Council of Medical Research (ICMR) National Ethical Guidelines (2017), and COREQ/SRQR standards for qualitative research reporting. We maintained strict confidentiality of all de‑identified interview data; in line with institutional policy, participants were informed that de‑identified study records might be inspected by authorised ethics or institutional oversight bodies (for example, the Institute’s ethics committee or designated regulators) solely for audit or monitoring purposes, and no identifying information was shared with any external agency during or after the study.

### Data analysis

We analysed the data using reflexive thematic analysis, following Braun and Clarke’s six‑phase framework [42]. First, authors read the transcripts repeatedly and wrote short memos to familiarise themselves with the content and note initial ideas. We then generated initial codes inductively and deductively in line with the research questions, applying these codes to meaningful segments that captured staff experiences of telemedicine in maternal and reproductive care. Related codes were collated into provisional themes, which were reviewed against both the coded extracts and the full data set; during this stage, themes were refined, split, merged, or discarded to ensure they were coherent, internally consistent, and adequately supported by the data. Once a stable structure was reached, we defined and named the final themes—such as infrastructural constraints, workflow disruptions, and opportunities to strengthen telemedicine—and clarified the analytic boundaries between them. Finally, we organised the themes into a narrative account, selecting illustrative quotations that demonstrated variation within and across cadres and highlighted implications for practice and policy. Throughout the analytic process, the multidisciplinary author team engaged in regular discussions to examine alternative interpretations and reflect on their positionality, and this reflexive deliberation informed both theme development and final interpretation.

We complemented the reflexive thematic analysis with an AI‑assisted, supervised sentiment analysis of staff statements about telemedicine for maternal and reproductive care. De‑identified transcripts from all 23 interviews were uploaded on 13 December 2025 to Google NotebookLM (2025), which uses large language models to generate provisional sentiment labels (positive, negative, neutral) for user‑defined text segments. The model outputs were not treated as autonomous findings: two researchers independently reviewed each AI‑assigned label against the corresponding transcript segment, corrected misclassifications, and reached consensus on the final sentiment category, thereby implementing a human‑in‑the‑loop supervisory process. For each cadre (CHOs, MOs, administrative doctors, IT staff), we then calculated, as simple descriptive statistics, the proportion of sentiment‑labelled segments (i.e. coded statements/tokens, not individual participants) that were positive, negative, or neutral. These distributions allowed us to descriptively quantify variation in perceived access, workload, and clinical quality across staff groups (e.g. “AI‑assisted sentiment analysis quantified the share of positive versus negative statements made by CHOs about tele‑ANC”). We did not conduct formal statistical tests, and we interpret these proportions as exploratory indicators that complement, rather than replace, the qualitative themes. All transcripts were de‑identified prior to upload and processed on secure, password‑protected accounts accessible only to the research team, in line with ICMR guidelines on privacy and data protection.

## Results

**Fig. 2:**
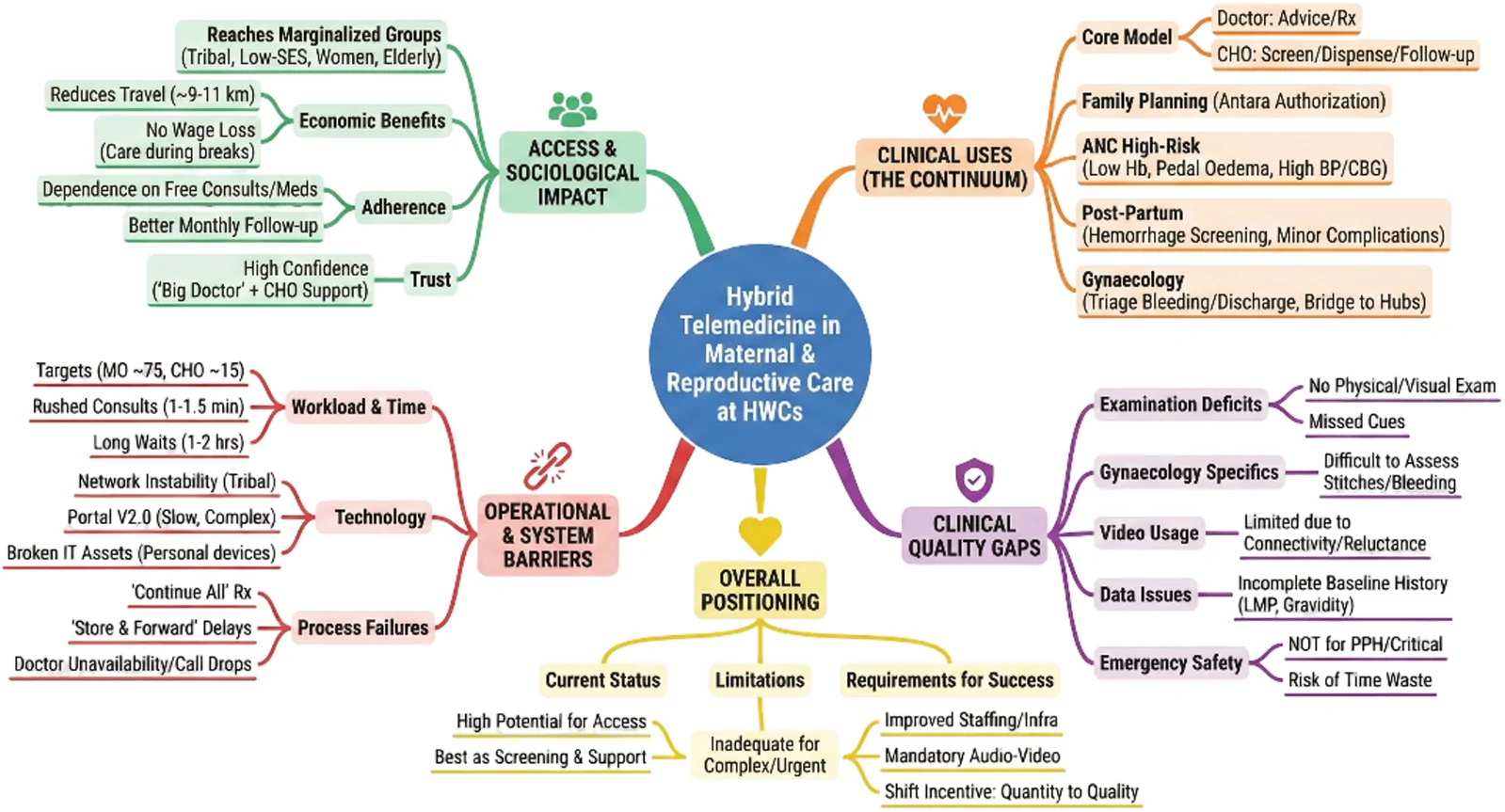
Emerged themes about hybrid telemedicine in maternal and reproductive care at HWCs

Throughout the analysis of interviews with CHOs, Medical Officers (MOs), and administrative staff, we identified geographical and socio-economic barriers to healthcare access. However, the analysis also highlighted ongoing issues with both operational and clinical quality, especially significant in the sensitive area of reproductive health. The derived Themes were showed in Fig. 2 and Table 1. For additional insights into the dynamics, please consult Supplementary Table S3.

**Table 1:** Themes and sub‑themes on hybrid telemedicine for maternal and reproductive healthcare in rural West Bengal

Theme	Sub-themes	Brief description
1. Sociological impact and enhancement of access to care	Access for marginalised groups; Economic benefit; Perceived enhanced trust; Increased adherence and reduced loss to follow-up	Telemedicine reduces distance, cost, and time barriers for rural, low-income women, increases trust in HWCs and remote doctors, and improves follow-up and continuity of care.
2. Clinical integration across the reproductive continuum	Family planning; Antenatal risk management; Postpartum support	Telemedicine supports safer contraceptive provision, co-management of high-risk pregnancies, and follow-up for minor postpartum issues within a CHO-assisted hybrid model.
3. Operational and systemic barriers to quality tele-consultation care	Process failures and accountability gaps; Workload and time pressure; Technological constraints	Doctor unavailability, long waits, high quantitative targets, network instability, and portal complexity disrupt teleconsultations and reduce the quality of care.
4. Clinical quality compromises in maternal assessment via hybrid telemedicine	Limits of remote gynaecological assessment; Examination deficits; Low audio-video use; Data and training gaps	Restricted physical examination, under-used audio-video, incomplete clinical information, and limited training create risks of superficial or inappropriate care for complex maternal and gynaecological cases.

### Theme 1: Sociological impact and enhancement of access to care

#### Sub-theme 1.1: Access for marginalized groups

The sociological and operational context of the hybrid telemedicine model reveals its impact on reducing barriers to healthcare access. Data confirms that the primary beneficiaries are those who are historically marginalised, specifically females, who belong to low socio-economic backgrounds. These populations are highly dependent on the free medication provided at the Health and Wellness Centre (HWC) (Additional file 3).

We generally see a higher number of elderly and female patients here, […] primarily economically disadvantaged. (Participant 12; CHO, 26–30 years, Female)

#### Sub-theme 1.2: Economic benefit

The hybrid operational model, which involves the doctor providing online prescriptions while the CHO delivers physical services, reduces the burden associated with healthcare seeking. Patients are relieved of the cost, time, and physical difficulty associated with traveling to distant referral hospitals. For pregnant women, who often lack guardians to accompany them, access to the HWC ensures regular medication. In our setting, telemedicine reduced the need for travel to higher‑level facilities but did not replace in‑person visits altogether, as patients still had to attend the Health and Wellness Centre to be assessed by the CHO and receive medicines.

Even if we advise them to go to the hospital, they are reluctant […] because of […] the cost of treatment, and the pressure of losing a day’s wages. Telemedicine saves their time and expenses. (Participant 12; CHO, 26–30 years, Female)

#### Sub-theme 1.3: Perceived enhanced trust

Patient satisfaction was also enhanced due to the telemedicine process, as they received consultations from senior or specialized doctors. Patients expressed being “very happy” and gained confidence from the knowledge that the CHO was consulting the “big doctor” on their behalf.

When this whole process is completed properly, the patients are very happy, and it gives us mental satisfaction too. Patients are quite happy to get the opportunity to speak directly with the doctor. (Participant 9; CHO, 46–50 years, Female)

This gives the patients a lot more confidence that, “Yes, the big doctor has seen me and prescribed the medicine; from now on, I’ll take the medicine from the centre.” Patients also come for regular medicine thereafter. (Participant 8; CHO; 36–40 years, Female)

#### Sub-theme 1.4: Increased adherence and reduced loss to follow-up

Patient satisfaction plays a crucial role in increasing the number of patients for telemedicine care and improving adherence to follow-up schedules. The telemedicine‑enabled HWC model strengthened continuity of care by allowing CHOs to track patients locally and conduct timely follow‑up, which staff felt noticeably reduced patients dropping out of treatment compared with earlier paper referrals. Participants repeatedly contrasted the current system with pre‑telemedicine practice, noting that women who previously disappeared between referrals and follow‑up visits were now more likely to return for scheduled care. By solving the problem of patients dropping out of care for their chronic diseases, the system is also simultaneously ensuring a safer and more continuous pathway for their reproductive health needs.

Earlier, […] patient […] treatment halfway. But now […] through tele-consultation […] follow-up patients […] has significantly increased. (Participant 12; CHO, 26–30 years, Female)

### Theme 2: Clinical integration across the reproductive continuum

Across family planning, antenatal, and postpartum care, telemedicine was perceived as clinically effective because it enabled rapid access to specialist guidance that CHOs could not otherwise obtain and helped maintain continuity of care across visits. Staff described the hybrid model as valuable not simply for creating a digital link, but because it allowed key reproductive health decisions to be completed at the HWC itself, reducing drop‑outs from repeated referrals to distant hospitals and sustaining follow‑up and ongoing treatment over time.

Telemedicine was seen as a crucial tool for integrating and enhancing reproductive healthcare across the continuum of services, serving as a link between primary care providers and specialised physician guidance in a hybrid care setting. This integration was evident across family planning, antenatal risk management, and post‑partum support.

A normal or cesarean delivery might not be feasible by telemedicine, but providing […] essential care […] can be considered a hybrid mode. A doctor prescribes […] online, whereas CHO […] providing the services physically. (Participant 21; Administrative officer; 51–55 years, Male)

In family planning and pre‑pregnancy care, telemedicine was essential for safely administering the Antara injectable contraceptive, as mandatory tele‑consultation allowed doctors to review vital signs (such as blood pressure and haemoglobin) and screen for contraindications like hypertension before authorising the injection. Staff emphasised that this digital step removed a major barrier, because women previously abandoned treatment when asked to travel solely to a PHC for a prescription.

We cannot give the ‘Antara injection’ without a doctor’s prescription. Earlier, patients had to be sent to the PHC (Primary Health Centre) for this. Due to this, many patients would abandon treatment midway and stop coming. This problem has been solved through telemedicine. (Participant 10; CHO, 36–40 years, Female)

During antenatal care and high‑risk pregnancy management, telemedicine filled critical clinical gaps by allowing CHOs to consult specialists immediately when complications were detected. Mandatory tele‑consultations for pregnant women facilitated rapid access to the Gynaecology and Obstetrics hub for high‑risk cases and helped ensure that referrals were taken seriously at higher‑level facilities.

Suppose a mother […] in her 2nd check-up […] low haemoglobin or swelling of the foot or high CBG (Capillary Blood Glucose) or high pressure, […]. Telemedicine is bridging the gap. The doctor […] give[es] the prescription […] […] investigation. (Participant 7; CHO. 36–40 years, Female)

When the patient went to the hospital, they initially didn’t give it much importance. However, when they saw the tele-consultation prescription from the patient, they considered the matter seriously. (Participant 12; CHO, 26–30 years. Female)

The application of telemedicine also extended into post‑partum support for issues such as post‑haemorrhage, abnormal blood pressure, or minor infant problems, reinforcing continuity before, during, and after pregnancy. Administrative staff described telemedicine as a screening and decision‑support tool that helped distinguish cases manageable with general care at the HWC from those requiring higher‑level referral.

I believe that telemedicine can be used as a screening method before, during, or after pregnancy. It will work as a support system. Through this, it can be said, “Yes, these […] will be fine with general care,” or “No, […] showi[ing] at a higher centre”. (Participant 19; Administrative officer; 31–35 years Male)

### Theme 3: Operational and systemic barriers to quality tele-consultation care

The integration of telemedicine aims to improve clinical effectiveness and increase access to quality healthcare. However, the successful execution of this core objective is frequently compromised by the effect of operational and systemic failures. These barriers, extended from personnel availability, quantitative pressure, and technical infrastructure, actively undermine the delivery of high-quality clinical care, often negating the very purpose of the tele-consultation model. (Additional file 3)

#### Sub-theme 3.1: Process failure and accountability gaps

One of the most immediate and critical barriers reported by CHOs was that, despite the programme’s aim of improving access to physicians, they often experienced doctors being unavailable at the time of consultation, leading to prolonged waits and, at times, incomplete tele‑visits. CHOs described recurrent ‘connection friction’—an analytic term we use to denote calls showing as busy, going unanswered, or being denied while patients waited ‘half an hour to one hour’ in the hope of speaking to a doctor. (Additional file 3)

When a call is made, it shows busy […] no one picks up […] or denied. […] Patients […] wait […] half an hour or an hour to talk to the doctor. (Participant 9; CHO, 46–50 years, Female)

These disruptions, combined with reports of superficial prescriptions, were perceived as raising questions about clinical accountability:

The sugar level might be high, and the doctor, […] simply writes “Continue All.” […] This raises a huge question about the quality of service. (Participant 14; CHO, 26–30 years, Female)

#### Sub-theme 3.2: Workload and time

Furthermore, the quality of care is systematically degraded by the imposition of high quantitative targets. The mandated volume—10 to 15 cases per day for CHOs and a upto 75 cases per day for Medical Officers (MOs)—creates intense pressure on staff. This pressure forces a detrimental focus on quantity over quality. CHOs have stated that the need to meet these indicators drastically shortens consultation times, often completing cases in less than 1 to 1.5 min. A direct consequence of this target pressure and the difficulty in establishing a connection is the practice of “Store and Forward,” where digital consultations and prescriptions are completed *after* the patient has already left due to the rush, requiring a subsequent call-back—a clear operational failure.

I have often seen that the quality of the prescriptions is […] not very good, […] the effort is to complete the work quickly, which […] leads to more emphasis on Quantity than Quality. (participant 5; CHO, 26–30 years, Female)

Is it right to set ‘targets’ for telemedicine […]? […] There is a tendency to quickly listen to the patient […] and then complete the cases later […] after all the patients leave […] due to […] targets. […] If no target is set, the quality […] significantly increases. (Participant 6; CHO, 26–30 years, Female)

#### Sub-theme 3.3: Technology

Technical and infrastructural deficiencies serve as foundational systemic barriers. Network instability is repeatedly cited as a “big challenge,” indicating telemedicine “almost impossible” in crucial remote outreach areas (where CHO visits and provide telemedicine services at doorstep through laptop or mobile) where the service is most needed. This core technical failure directly interrupts service continuity. Adding to this is the complexity and friction of the operational portal. (Additional file 4) The new V2.0 portal, with its “numerous AI-generated questions,” requires a lengthy 15–20 min for registration, significantly exacerbating patient waiting and frustration. When technical equipment fails, such as with broken laptops or printers, staff are forced to use personal phones—a technically “not allowed” but necessary improvisation to maintain the semblance of continuity, underscoring the gap between systemic regulation and operational reality.

When I am in the center network is stable but when […] outreach work […] network is a big challenge. […] Net (internet) connection is an issue […] so I am not able to do telemedicine there. (Participant 1; CHO, 31–35 years, Female)

Network issues are a major obstacle because the portal […] if the network speed is slow. […] [It] becomes difficult […] to meet their targets […] network problem is very significant. (Participant 22; IT Staff, 26–30 years, Female)

The efficacy of the tele-consultation model is severely curtailed by a complex interplay of systemic barriers. From the unavailability of doctors and the subsequent negation of the consultation’s purpose, to the overwhelming quantitative pressure that forces a compromise on quality, and finally, to the instability of infrastructure and complexity of the operating portal—these operational and systemic failures consistently undermine the delivery of quality clinical care, necessitating a critical re-evaluation of current implementation strategies.

### Theme 4: Clinical quality compromises in maternal health assessment via hybrid telemedicine

The integration of telemedicine into maternal health services, particularly through a hybrid model, presents a dilemma between efficiency and clinical safety. The most important concern arising from this model is significant compromise in adequate clinical assessment remotely, which raises questions about both the safety and effectiveness of the care provided.

#### Sub-theme 4.1: Gynaecologist deficit

A core limitation of the hybrid model is the structural inadequacy of remote gynaecological assessment. Both Medical Officers (MOs) and CHOs consistently endorsed the superiority of physical consultation, deeming it “always better”. A connection to a specialty hub, such as Gynae, is often initiated when the specialist simply writes, “refer to gyne OPD.” This redirection not only questions the quickness and convenience of the tele-consultation but also transforms the service into just a referral mechanism rather than a site of primary clinical resolution.

Also, after a day’s work, […] no energy left […] to do telemedicine. […] We need to rely on Didi’s (CHO) words. […] [It] is not right to treat without knowing or understanding whether the patient has any other complications. (Participant 19; Administrative Officer, 31–35 years, Male)

#### Sub-theme 4.2: Examination deficit

Remote assessment through telemedicine might compromise care. This amplifies when medical personnel are given quantitative pressure to meet target consultation numbers, and it leads to instances of reduced care quality. Prescription errors are a documented consequence, with CHOs reporting that doctors, in their hurry, sometimes ordered “Continue All” even when a patient’s vitals, such as dangerously high blood sugar, clearly mandated a change in medication. One CHO asserted, *“raise a huge question about the quality of service.”* Furthermore, information deficits on the CHO side exacerbate the problem. MOs noted that CHOs often lacked the necessary training to provide essential baseline data for complex cases, such as the patient’s date of menstrual cycle, gravidity, and parity.

As a doctor […] training we were given was to treat the patient after seeing them, i.e., treating after a physical examination. This kind of training [for telemedicine] was not given. (Participant 19; Administrative Officer, 31–35 years, Male)

I have had the experience that a patient may not be taking any medicine, but the doctor has written ‘Continue all’ on the prescription. […] I feel they wrote it without even seeing my patient’s case. […] Before, when I used to do about 40–45 consultations, half the prescriptions were like this. (Participant 5; CHO, 26–30 years, Female)

There is a need for training of us doctors as well as training of CHOs. Maybe training or a workshop where both the parties are there where we can inform or guide the CHOs that you need to provide this much of the information and in this way, you need to communicate. (Participant 16; Medical Officer, 36–40 years, Male)

#### Sub-theme 4.3: Video usage

The clinical process is severely hampered by the frequent lack of reliable audio/video contact, forcing the doctor to rely entirely on the CHO’s written or verbal report. This reliance is particularly unstable in reproductive health, where slight yet crucial signs are often missed. For treating gynaecological problems video calls are essential and relying solely on written communication was not enough, explicitly confirmed by the participants.

If they express their problem even through tele and speak I can ask related questions, […] even if I am not touching the patient. If I cannot see the patient then I have to wholly rely on what CHO wrote to me. If I can see and speak with the patient then the service will be more fruitful. (Participant 16; Medical Officer, 36–40 years, Male)

#### Sub-theme 4.4: Data issues

For effective treatment, the medical history or current medical information of the patient is necessary, but in some cases, doctors had to treat with inadequate information, which raises concern. CHOs specifically reported struggling to accurately describe sensitive issues such as white discharge, the nature of post-delivery infection or dryness, or the precise appearance of a C-section wound.

The details of baseline, the date of menstrual cycle, patients gravida and parity were missing. CHOs are lacking to provide adequate information and this needs to be corrected. (Participant 16; Medical Officer, 36–40 years, Male)

There are many problems that cannot be shown directly, and that’s why it is difficult to describe them accurately to the doctors. For example, white discharge, accumulation of pus, or post-delivery vaginal infection or dryness […] the exact condition, colour, […] cannot be easily expressed […] similarly condition of the wound or stitches after a Caesarean section or other surgeries, […] cannot be properly explained. (Participant 2; CHO, 36–40 years, Female)

The main understanding from the health staff was the consensus that telemedicine is simply not the right tool for handling critical emergencies, like a high glucose crisis, heavy vaginal bleeding, or severe loose motion. The CHOs are trained to refer to these cases immediately, but the necessary step of trying to connect with the tele-doctor often causes a slight, but crucial, delay. This highlights that telemedicine is most valuable as a supportive resource or a helpful screening step, rather than a comprehensive treatment solution for complex maternity situations.

### Sentiments of informants towards access and quality of hybrid telemedicine in maternal and reproductive health

**Fig. 3:**
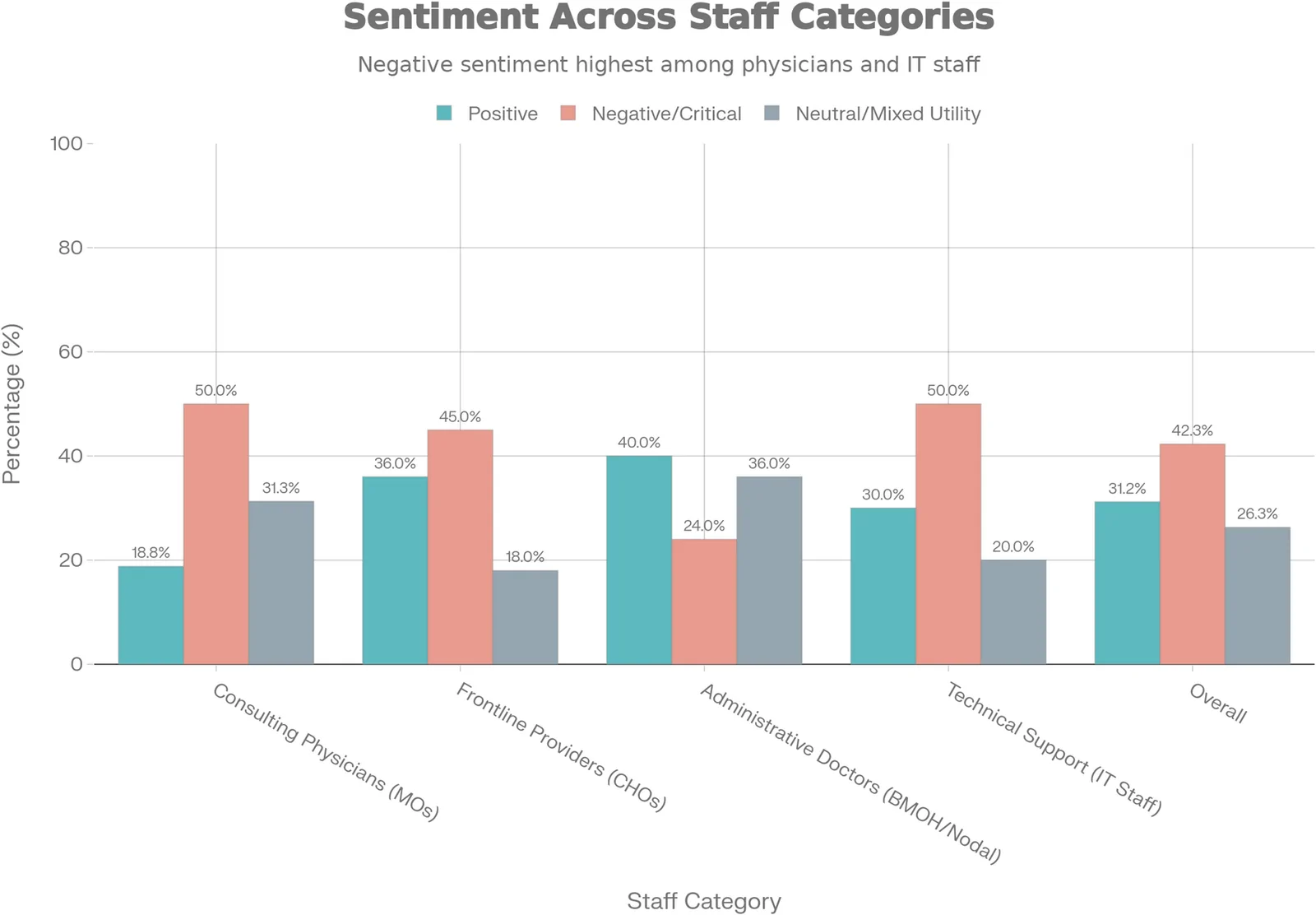
Clustered bar chart showing the distribution of sentiment‑labelled segments across staff categories

**Fig. 4:**
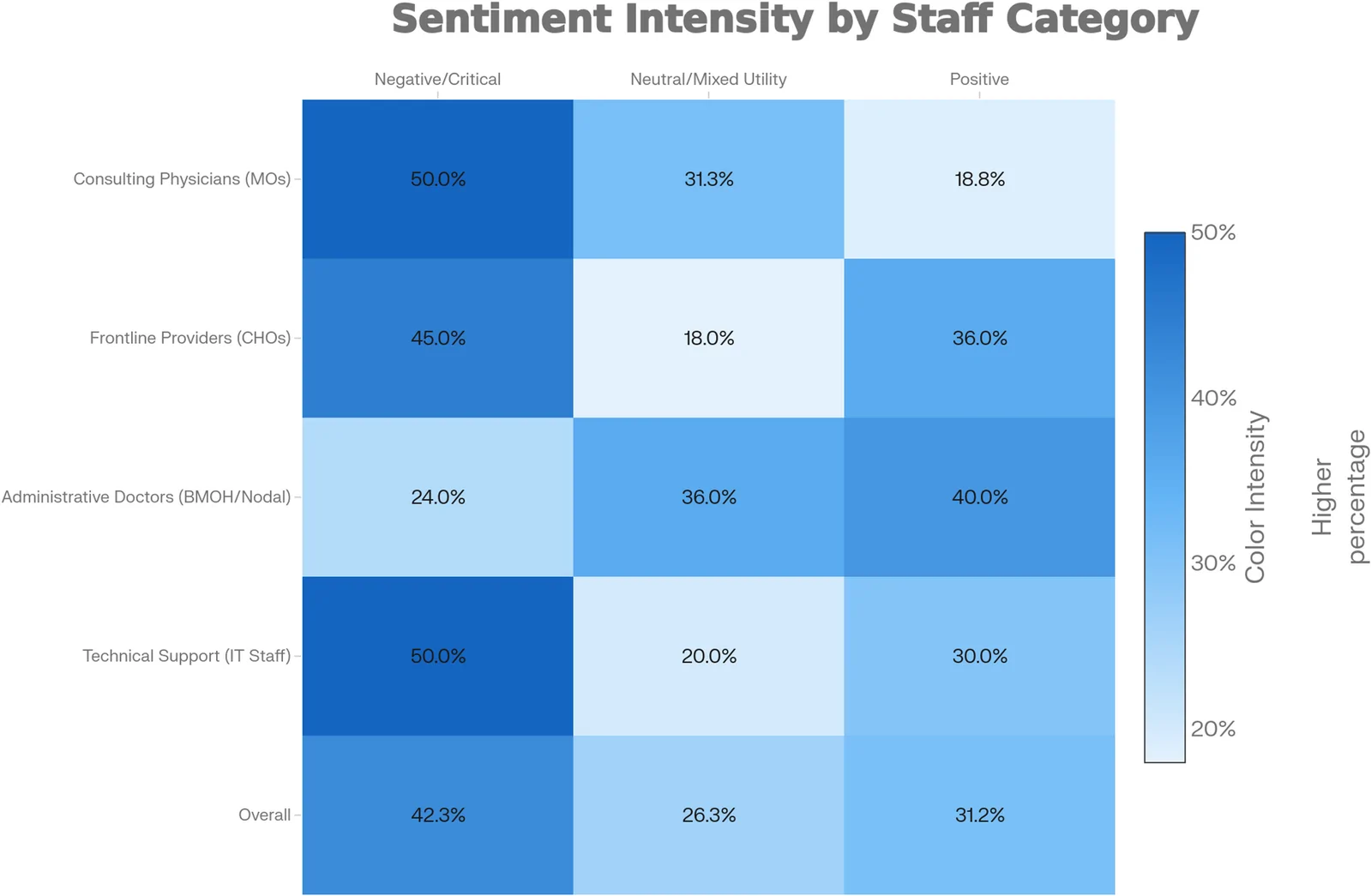
Sentiment intensity of coded segments across groups of health personnel

Structured analysis of sentiment‑labelled statements revealed clear differences in how staff cadres viewed the hybrid telemedicine model. Consulting physicians (MOs) showed the highest share of negative or critical sentiment (around half of their statements), driven mainly by concerns about clinical risk from very short teleconsultations and the inability to perform essential physical examinations for complex maternal cases. Frontline CHOs also expressed substantial critical sentiment (about 45%), emphasising long waiting times for doctors (up to one or two hours), call denials, and technical friction such as lengthy portal registrations. In contrast, administrative doctors (BMOH/nodal) had the highest positive sentiment (about 40%), highlighting system‑level benefits like reduced travel for women, decongestion of higher‑level facilities, and progress towards macro‑level public health goals. IT staff were predominantly negative (around 50%), pointing to recurrent network instability, inadequate technical manpower, and complex portal design as major operational problems. Taken together, these descriptive patterns—summarised in the sentiment table (Table S4) and Figs. 3 and 4— concur with the thematic findings and underscore a tension between access gains and perceived compromises in clinical quality, with ground‑level providers (CHOs and MOs) more critical about telemedicine’s functionality than administrative officials.

## Discussion

The integration of telemedicine into reproductive healthcare represents a significant shift, particularly in resource-constrained and geographically isolated regions. This model serves a critical role, utilizing technology to reduce access barriers while relying on frontline CHOs to provide the essential physical link [31, 43, 44]. The successful implementation of this hybrid system, particularly in sensitive fields such as obstetrics and gynaecology, necessitates addressing current issues that compromise the quality and completeness of care [33, 45].

Telemedicine transforms the delivery of reproductive health services throughout the care continuum. It significantly broadens access to medical expertise across the pre-pregnancy, antenatal care (ANC), and postpartum phases [38, 46–48]. Within the domain of family planning, for example, Telemedicine makes it possible to remotely conduct the vital assessment of blood pressure and other vitals necessary to prescribe injectable contraceptives, such as the ‘Antara injection’, with the help of CHO, facilitating crucial consultations. Telemedicine plays a notable role in Antenatal Care (ANC) by enabling the effective management of complications detected by CHOs, such as high blood pressure or anaemia. This capability is critical because it directly connects high-risk mothers with specialist consultations [38, 40]. Telemedicine significantly improves the feasibility of essential doctor consultations for mothers to receive the required four ANC check-ups. It provides a convenient method for minor post-partum and gynaecological concerns, including issues ranging from mild bleeding to managing post-natal complications. However, a crucial principle is that telemedicine serves primarily as a screening and support system-it is not a replacement for comprehensive physical treatment, particularly in complex or critical cases [49].

The current hybrid-assisted telemedicine model, similar to other Indian implementations of eSanjeevani and innovative assisted teleconsultation initiatives, relies on the CHO as the crucial physical link between remote physicians and patients. In this system, the doctor provides an online prescription, while the CHO operationalizes care on the ground by managing patient data, recording vital signs, offering basic symptomatic or intermittent relief to patients awaiting consultation, and dispensing prescribed medicines. The CHO’s role further extends to tracking patients and conducting follow-up, thereby helping continuity of care beyond the teleconsultation episode and supporting ongoing management [50, 51].

Telemedicine has had a profoundly positive impact on patient access and experience. It effectively overcomes significant access barriers by reducing the financial costs, time, and stress associated with travel. This is particularly beneficial for economically vulnerable individuals, the elderly, pregnant mothers, and those residing in remote, rural areas with inadequate road networks [22, 40, 52]. The convenience of attending an appointment during a work break or securing regular medication even without a caregiver represents a major advantage. As a result, patient satisfaction and confidence often improve, a finding consistent with other studies that have captured patient satisfaction with telemedicine [53, 54]. Direct virtual consultations with a physician build trust in the Health and Wellness Centre (HWC) and motivate patients to use the service and recommend it to others. Taken together, these findings suggest that when adequately supported, the CHO-assisted hybrid model can act as a digital umbilical, sustaining a continuous, two-way flow of reproductive care between rural women and distant specialists across the full maternal continuum.

Despite these benefits, the implementation of telemedicine in obstetrics and gynaecology faces substantial operational and clinical limitations. A primary challenge is poor connectivity to specialized hubs, often leading specialists to simply recommend “refer to gynaecology OPD,” which undermines the teleconsultation’s intent. More critically, remote assessments are constrained by inadequate physical examinations for issues like vaginal bleeding, discharge, or post-operative wounds such as C-section stitches [55]. Video consultations, which are essential for gynaecological evaluations, are frequently affected by physician reluctance and network instability [51, 56]. Consequently, experts agree that telemedicine is inappropriate for emergencies like heavy vaginal bleeding, where protocols mandate immediate in-person referral rather than lengthy virtual attempts [45].

These clinical limitations are also increased by systemic constraints. Mandatory daily consultation targets for CHOs and doctors prioritize service volume over quality, leading to superficial practices that compromise thorough patient care. This emphasis on quantity drives providers toward speed-driven shortcuts, such as issuing generalized “Continue All” prescriptions despite evident needs for adjustment (e.g., elevated blood glucose levels). Such target-driven performance indicators risk generating a ‘Cobra Effect’, where well‑intentioned incentives produce counterproductive provider behaviours [57]. These metrics inadvertently promote target-oriented behaviour over the comprehensive, patient-centred care that the telemedicine initiatives intend [58].

Recurrent information deficits during teleconsultations can affect the quality of care, with CHOs sometimes omitting key baseline details, such as the last menstrual period, gravidity, and parity, that are essential for accurate clinical assessment and safe prescribing decisions, including injectable contraceptives (‘Antara injections’). This highlights the need for structured capacity-building, including targeted training and hands-on workshops for both doctors and CHOs. It will standardize clinical history-taking, documentation, and communication protocols during teleconsultations. In addition, persistent infrastructural and operational constraints, such as doctor availability, prolonged waiting times, unstable network connectivity, and recurring technical faults with telemedicine equipment delay timely care for pregnant women, who often cannot afford extended waiting or repeated visits. Similar longstanding telemedicine implementation challenges have been noted in the broader literatures [33, 59–62].

Beyond technical and workflow barriers, our findings also suggest a subtle ‘deshouldering’ of responsibility between frontline CHOs and remote doctors, with each side sometimes perceiving that the other should carry the primary clinical and operational burden of teleconsultations. This dynamic appeared to undermine ownership of the intervention and contributed to episodes of denied calls, superficial prescriptions, and frustration among CHOs, despite broad recognition that the model improves access for patients. Implementer acceptability therefore emerges as a critical determinant of telemedicine quality: when providers are unconvinced about the model or feel over‑burdened by targets and connection failures, their engagement with the system declines, which in turn threatens the consistent delivery and clinical safety of the intervention.

Clinical staff sentiments highlight a tension between improved access through telemedicine and maintaining clinical quality in maternal care. Administratively, tele‑ANC and hybrid telemedicine models are valued for reducing women’s travel burden and improving access, particularly in remote or underserved areas. This aligns with evidence from Indian and international ANC programs. However, studies on telemedicine systems in India also pointed out that when administrators emphasize consultation volume under conditions of weak IT/network infrastructure, this can increase provider workload, undermine clinical decision‑making, and risk superficial prescriptions. Policy refinement should therefore maintain administrative goals of expanded coverage and reduced travel by investing in infrastructure and a robust quality-assurance process to safeguard maternal health [63–66].

This study has several limitations. First, the sample comprised 23 purposively selected staff members from two districts of West Bengal, which limits the generalisability of our findings to other Indian states or health‑system contexts. Second, our use of AI‑assisted sentiment analysis was exploratory; the reported percentages describe the distribution of sentiment‑labelled statements rather than statistically powered estimates, and we did not perform formal significance testing. Third, the telemedicine programme and digital infrastructure in West Bengal may differ from those in other settings, so our findings should be interpreted as context‑specific insights that nonetheless highlight broader issues for hybrid telemedicine in maternal and reproductive health. However, because the participating cadres share comparable training, roles, and exposure to the state telemedicine platform, these qualitative insights are likely to be informative for similar implementation settings beyond the two study districts. Finally, although similar telemedicine services are available through national programmes, allowing some cautious inference beyond this setting, these parallels require further empirical exploration.

## Conclusion

Hybrid telemedicine at Health and Wellness Centres can substantially narrow access gaps in maternal and reproductive healthcare for rural women when CHOs are empowered as skilled on‑site assessors and consistent remote clinical support is available. At the same time, our findings show that systemic weaknesses—doctor unavailability, rigid daily targets, incomplete clinical information, and unstable digital infrastructure—can undermine clinical quality and safety, especially for complex maternal and gynaecological conditions. To realise the “digital umbilical” potential of this model, telemedicine should be explicitly positioned as a triage, screening, and support layer that helps identify high‑risk women early, initiate basic management, and channel them efficiently to higher‑level facilities, rather than as a substitute for comprehensive in‑person care. Policymakers should therefore rebalance performance metrics away from consultation counts towards quality‑of‑care indicators, invest in joint training for CHOs and MOs on maternal teleconsultations and clinical decision‑making, and ensure reliable connectivity and user‑friendly platforms so that remote consultations are both technically robust and clinically meaningful. Strengthening the CHO’s physical support role and safeguarding the integrity of the remote consultation are both essential if hybrid telemedicine is to function as a safe and effective triage system within the reproductive health continuum.

## Supplementary Information

Below is the link to the electronic supplementary material.

Supplementary Material 1

Supplementary Material 2

Supplementary Material 3

Supplementary Material 4

## Data Availability

The datasets and interview transcripts generated and/or analysed during the current study are not publicly available due to participant confidentiality but are available from the principal investigator, Sibasis Mandal (email: mandal.sibasis@gmail.com), upon reasonable request. All data are securely stored and will be permanently destroyed five years after study completion.

## Abbreviations

ANC: Antenatal Care
BMOH: Block Medical Officer of Health
CHO: Community Health Officer
HWC: Health and Wellness Centre
IDI: In-Depth-Interview
IT: Information Technology
MO: Medical Officer
OPD: Out Patient Department
PHC: Primary Health Centre

## References

[1] Cresswell JA, Alexander M, Chong MYC, Link HM, Pejchinovska M, Gazeley U, et al. Global and regional causes of maternal deaths 2009–20: a WHO systematic analysis. Lancet Glob Health. 2025;13(4):e626–34. 10.1016/S2214-109X(24)00560-6. PMC11946934 40064189

[2] Service coverage index. and components (SDG 3.8.1) [Internet]. [cited 2025 Dec 10]. Available from: https://www.who.int/data/gho/data/themes/topics/service-coverage.

[3] van Duinen AJ, Adde HA, Fredin O, Holmer H, Hagander L, Koroma AP, et al. Travel time and perinatal mortality after emergency caesarean sections: an evaluation of the 2-hour proximity indicator in Sierra Leone. BMJ Glob Health. 2020;5(12):e003943. 10.1136/bmjgh-2020-003943. PMC7754652 33355267

[4] Kelly C, Hulme C, Farragher T, Clarke G. Are differences in travel time or distance to healthcare for adults in global north countries associated with an impact on health outcomes? A systematic review. BMJ Open. 2016;6(11):e013059. 10.1136/bmjopen-2016-013059. PMC5178808 27884848

[5] Nemet GF, Bailey AJ. Distance and health care utilization among the rural elderly. Soc Sci Med. 2000;50(9):1197–208. 10.1016/S0277-9536(99)00365-2. 10728841

[6] McHenry RD, Moultrie CEJ, Corfield AR, Lone NI, Mitchell R, Mackay DF, et al. Geographic isolation, rurality, and outcomes after critical illness: a retrospective cohort study of emergency admissions to critical care units in Scotland 2010–2021. Br J Anaesth. 2025;135(3):632–41. 10.1016/j.bja.2025.05.019. 40562632

[7] Doogan NJ, Roberts ME, Wewers ME, Tanenbaum ER, Mumford EA, Stillman FA. Validation of a new continuous geographic isolation scale: A tool for rural health disparities research. Soc Sci Med. 2018;215:123–32. 10.1016/j.socscimed.2018.09.005. PMC6182768 30227352

[8] Ali B, Chauhan S. Inequalities in the utilisation of maternal health Care in Rural India: Evidences from National Family Health Survey III & IV. BMC Public Health. 2020;20(1):369. 10.1186/s12889-020-08480-4. PMC7082939 32197599

[9] Barrett B, Charles JW, Temte JL. Climate change, human health, and epidemiological transition. Prev Med. 2015;70:69–75. 10.1016/j.ypmed.2014.11.013. PMC4342988 25434735

[10] Henderson JW, Taylor BA. Rural isolation and the availability of hospital services. J Rural Stud. 2003;19(3):363–72. 10.1016/S0743-0167(03)00007-X.

[11] Chen L, Chen T, Lan T, Chen C, Pan J. The Contributions of Population Distribution, Healthcare Resourcing, and Transportation Infrastructure to Spatial Accessibility of Health Care. Inq J Health Care Organ Provis Financ. 2023;60:00469580221146041. 10.1177/00469580221146041. PubMed PMID: 36629371. PMC9837279 36629371

[12] Kruk ME, Gage AD, Arsenault C, Jordan K, Leslie HH, Roder-DeWan S, et al. High-quality health systems in the Sustainable Development Goals era: time for a revolution. Lancet Glob Health. 2018;6(11):e1196–252. 10.1016/S2214-109X(18)30386-3. PMC7734391 30196093

[13] Abernethy A, Adams L, Verily, National Academy of Medicine, Barrett M, ResMed, et al. The promise of digital health: then, now, and the future. NAM Perspect. 2022;6(22). 10.31478/202206e. PMC9499383 36177208

[14] Erku D, Khatri R, Endalamaw A, Wolka E, Nigatu F, Zewdie A, et al. Digital Health Interventions to Improve Access to and Quality of Primary Health Care Services: A Scoping Review. Int J Environ Res Public Health. 2023;20(19):6854. 10.3390/ijerph20196854. PMC10572344 37835125

[15] Patrick K, Hekler EB, Estrin D, Mohr DC, Riper H, Crane D, et al. The Pace of Technologic Change: Implications for Digital Health Behavior Intervention Research. Am J Prev Med. 2016;51(5):816–24. 10.1016/j.amepre.2016.05.001. 27745681

[16] Michie S, Yardley L, West R, Patrick K, Greaves F. Developing and evaluating digital interventions to promote behavior change in health and health care: recommendations resulting from an international workshop. J Med Internet Res. 2017;19(6):e232. 10.2196/jmir.7126. PMC5509948 28663162

[17] Barbosa W, Zhou K, Waddell E, Myers T, Dorsey ER. Improving Access to Care: Telemedicine Across Medical Domains. Annu Rev Public Health. 2021;42(1):463–81. 10.1146/annurev-publhealth-090519-093711. 33798406

[18] Yeung AWK, Torkamani A, Butte AJ, Glicksberg BS, Schuller B, Rodriguez B, et al. The promise of digital healthcare technologies. Front Public Health. 2023;11:1196596. 10.3389/fpubh.2023.1196596. PMC10562722 37822534

[19] McCarthy D. The virtual health economy: telemedicine and the supply of primary care physicians in rural America. Am J Law Med. 1995;21(1):111–30. PubMed PMID: 7573080. 7573080

[20] COMMITTEE ON PEDIATRIC WORKFORCE, Marcin JP, Rimsza ME, Moskowitz WB. The Use of Telemedicine to Address Access and Physician Workforce Shortages. Pediatrics. 2015;136(1):202–9. 10.1542/peds.2015-1253 . PubMed PMID: 26122802. 26122802

[21] FangML, Siden E, Korol A, Demestihas MA, Sixsmith J, Sixsmith A. A scoping review exploration of the intended and unintended consequences of eHealth on older people: a health equity impact assessment. Hum Technol. 2018;297–323. https://ht.csr-pub.eu/index.php/ht/article/view/206/185.

[22] Mulvale G, Moll S, Miatello A, Robert G, Larkin M, Palmer VJ, et al. Codesigning health and other public services with vulnerable and disadvantaged populations: Insights from an international collaboration. Health Expect. 2019;22(3):284–97. 10.1111/hex.12864. PMC6543156 30604580

[23] Lopreite M, Zhu Z. The effects of ageing population on health expenditure and economic growth in China: A Bayesian-VAR approach. Soc Sci Med. 2020;265:113513. 10.1016/j.socscimed.2020.113513. 33199036

[24] Lopreite M, Misuraca M, Puliga M. An analysis of the thematic evolution of ageing and healthcare expenditure using word embedding: A scoping review of policy implications. Socioecon Plann Sci. 2023;87:101600. 10.1016/j.seps.2023.101600.

[25] WHO Global Observatory for eHealth. Telemedicine: opportunities and developments in member states: report on the second global survey on eHealth [Internet]. Geneva: World Health Organization; 2010 [cited 2025 Jan 29]. Available from: https://iris.who.int/handle/10665/44497.

[26] Russo JE, McCool RR, Davies LVA, Telemedicine. An Analysis of Cost and Time Savings. Telemed E-Health. 2016;22(3):209–15. 10.1089/tmj.2015.0055. 26305666

[27] Mishra SK, Kapoor L, Singh IP. Telemedicine in India: current scenario and the future. Telemed J E-Health Off. J Am Telemed Assoc. 2009;15(6):568–75. 10.1089/tmj.2009.0059 . PubMed PMID: 19659413. 19659413

[28] Anand A, Trivedi NK, Gautam V, Arvindhan M. Infrastrucuture and systems of telemedicine. In: Choudhury T, Katal A, Um JS, Rana A, Al-Akaidi M, editors. Telemedicine: the computer transformation of healthcare [Internet]. Cham: Springer International Publishing; 2022. pp. 29–41. Available from: 10.1007/978-3-030-99457-0_3.

[29] Tripathi N, Parhad P, Garg S, Biswal SS, Ramasamy S, Panda A, et al. Performance of health and wellness centre in providing primary care services in Chhattisgarh, India. BMC Prim Care. 2024;25(1):360. 10.1186/s12875-024-02603-1. PMC11451184 39367295

[30] LahariyaC. Health & Wellness Centers to Strengthen Primary Health Care in India: Concept, Progress and Ways Forward. Indian J Pediatr. 2020;87(11):916–29. PubMed PMID: 32638338. 10.1007/s12098-020-03359-z. PMC7340764 32638338

[31] Sood S, Lal K, Bhatia M, Kapoor G, Singh S, Kaushish RK, et al. Adoption and utilization of India’s eSanjeevani national telemedicine service. Oxf Open Digit Health. 2025;3:oqaf025. 10.1093/oodh/oqaf025. PMC12558045 41158553

[32] Dastidar BG, Jani AR, Suri S, Nagaraja VH. Reimagining India’s national telemedicine service to improve access to care. Lancet Reg Health - Southeast Asia. 2024;30. 10.1016/j.lansea.2024.100480. PMC11422547 39323563

[33] Dutta S, Chakrabarti S, Basu M, Manna S. Challenges Faced by Community Health Officers and Beneficiary Satisfaction Related to Telemedicine Services at Health and Wellness Centers: A Mixed-method Study from a Block of West Bengal. Indian J Community Med. 2025;50(2):361–7. 10.4103/ijcm.ijcm_330_23. PMC12080889 40384836

[34] Telemedicine_Practice_Guidelines.pdf [Internet]. [cited 2025 Dec 10]. Available from: https://esanjeevani.mohfw.gov.in/assets/guidelines/Telemedicine_Practice_Guidelines.pdf

[35] Dinakaran D, Manjunatha N, Kumar CN, Math SB. Telemedicine practice guidelines of India, 2020: Implications and challenges. Indian J Psychiatry. 2021;63(1):97. 10. 4103/PSYCHIATRY.INDIANJPSYCHIATRY_476_20 PubMed PMID: 34083829. 10.4103/psychiatry.IndianJPsychiatry_476_20 PMC8106416 34083829

[36] Ministry of Health and Family Welfare, Government of India. Ayushman Bharat Health and Wellness Centres: Realizing Universal Health Care [Internet]. New Delhi: Press Information Bureau; 2021 Sep [cited 2025 Dec 13]. Available from: https://static.pib.gov.in/WriteReadData/specificdocs/documents/2021/sep/doc202192010.pdfBharat.

[37] Ministryof Health and Family Welfare, Government of India. Guidelines for Tele-Medicine Services in Ayushman Bharat- Health and Wellness Centres (HWCs) [Internet]. New Delhi: National Health Mission; [date unknown] [cited 2025 Dec 13]. Available from: https://nhsrcindia.org/sites/default/files/2021-06/GUIDELINES%20FOR%20TELEMEDICINES.pdf.

[38] Le Vance J, Gurney L, Morris RK, Morton VH. Telemedicine and remote monitoring advancements for obstetric antenatal care. Obstet Gynaecol Reprod Med. 2025;35(9):266–70. 10.1016/j.ogrm.2025.06.004.

[39] Young D, Schumm LP, McHugh A, Whitaker AK, Stulberg D. Rapid Innovation and Adaptation in Contraceptive Care Using Telemedicine: Evaluating Impact and Sustainability at Planned Parenthood of Illinois. Telemed J E-Health Off J Am Telemed Assoc. 2025;31(6):737–46. 10.1089/tmj.2024.0513 . PubMed PMID: 39982777. 39982777

[40] Bonifasius KIPG, Abdul Madjid O, Seno Adjie JM, Marceliano Rumopa HI. Effectiveness of the telemedicine approach on maternal health practices among pregnant women in rural areas. Indones J Obstet Gynecol. 2024;179–85. 10.32771/inajog.v12i3.2100.

[41] Districts having weak Health Infrastructure [Internet]. [cited 2025 Dec 15]. Available from: https://www.pib.gov.in/www.pib.gov.in/Pressreleaseshare.aspx?PRID=1539869

[42] Braun V, Clarke V. Using thematic analysis in psychology. Qual Res Psychol. 2006;3(2):77–101. 10.1191/1478088706qp063oa.

[43] Virag M, Kovacs R, Marovics G, Toth L, Sandor B, Voros P, et al. Bridging healthcare gaps through specialized mobile healthcare services to improve healthcare access and outcomes in rural Hungary. Sci Rep. 2025;15(1):12692. 10.1038/s41598-025-97447-9. PMC11993730 40221601

[44] Ezeamii VC, Okobi OE, Wambai-Sani H, Perera GS, Zaynieva S, Okonkwo CC, et al. Revolutionizing healthcare: how telemedicine is improving patient outcomes and expanding access to care. Cureus. 2024 Jul;5. 10.7759/cureus.63881. PMC11298029 39099901

[45] Medani IE, Hakami AM, Chourasia UH, Rahamtalla B, Adawi NM, Fadailu M, et al. Telemedicine in Obstetrics and Gynecology: A Scoping Review of Enhancing Access and Outcomes in Modern Healthcare. Healthcare. 2025;13(16):2036. 10.3390/healthcare13162036. PMC12385597 40868652

[46] Atkinson J, Hastie R, Walker S, Lindquist A, Tong S. Telehealth in antenatal care: recent insights and advances. BMC Med. 2023;21(1):332. 10.1186/s12916-023-03042-y. PMC10470141 37649028

[47] Tormen M, Toniolo B, Pecci L, Soraci G, Taliento C, Greco P, et al. Exploring the impact of integrating telehealth in obstetric care: A scoping review. Eur J Obstet Gynecol Reprod Biol. 2024;302:242–8. 10.1016/j.ejogrb.2024.09.031. 39332087

[48] Hawkins SS. Telehealth in the Prenatal and Postpartum Periods. J Obstet Gynecol Neonatal Nurs. 2023;52(4):264–75. 10.1016/j.jogn.2023.05.113. PMC10248753 37302795

[49] Haleem A, Javaid M, Singh RP, Suman R. Telemedicine for healthcare: Capabilities, features, barriers, and applications. Sens Int. 2021;2:100117. 10.1016/j.sintl.2021.100117. PMC8590973 34806053

[50] Jose AP, Kaushik A, Tange H, Van Der Weijden T, Pandey N, Sharma A, et al. Redesigning telemedicine: preliminary findings from an innovative assisted telemedicine healthcare model. BMC Prim Care. 2024;25(1):380. 10.1186/s12875-024-02631-x. PMC11515714 39443848

[51] BG D, AR J. Reimagining India’s National Telemedicine Service to improve access to care. Lancet Reg Health Southeast Asia. 2024;30:100480. 10.1016/j.lansea.2024.100480. PMC11422547 39323563

[52] Anawade PA, Sharma D, Gahane S. A comprehensive review on exploring the impact of telemedicine on healthcare accessibility. Cureus. 2024 Mar 12. 10.7759/cureus.55996. PMC11009553 38618307

[53] Nguyen M, Waller M, Pandya A, Portnoy J. A Review of Patient and Provider Satisfaction with Telemedicine. Curr Allergy Asthma Rep. 2020;20(11):72. 10.1007/s11882-020-00969-7. PMC7505720 32959158

[54] Gustke SS, Balch DC, West VL, Rogers LO. Patient Satisfaction with Telemedicine. Telemed J. 2000;6(1):5–13. 10.1089/107830200311806.

[55] Grimes CL, Balk EM, Dieter AA, Singh R, Wieslander CK, Jeppson PC, et al. Guidance for gynecologists utilizing telemedicine during COVID-19 pandemic based on expert consensus and rapid literature reviews. Int J Gynecol Obstet. 2020;150(3):288–98. 10.1002/ijgo.13276.

[56] Singh A, Agarwal A, Thakur G, Goel S, Singh M. Role of telemedicine in obstetrics and gynecology: an experience at tertiary care center. Expert Rev Med Devices. 2023;20(12):1251–6. 10.1080/17434440.2023.2264768. 37753857

[57] Blogger C, American Institute of Healthcare Compliance [Internet]. The cobra effect & enforcing compliance standards. 2024 Feb 16 [cited 2025 Dec 12]. Available from: https://aihc-assn.org/the-cobra-effect-enforcing-compliance-standards/.

[58] CholleteL, Harrison SG. Unintended Consequences: Ambiguity Neglect and Policy Ineffectiveness. East Econ J. 2021;47(2):206–26. . 021-00187-7 PubMed PMID: 33551514; PubMed Central PMCID: PMC7848876. 10.1057/s41302-021-00187-7. PMC7848876 33551514

[59] StanberryB. Telemedicine: barriers and opportunities in the 21st century. J Intern Med. 2000;247(6):615–28. PubMed PMID: 10886483. 10.1046/j.1365-2796.2000.00699.x. 10886483

[60] Hadian M, Jelodar ZK, Khanbebin MJ, Atafimanesh P, Asiabar AS, Dehagani SMH. Challenges of implementing telemedicine technology: a systematized review. Int J Prev Med. 2024;15. 10.4103/ijpvm.ijpvm_48_23. PMC10982727 38563036

[61] Adhikari P. Leveraging telemedicine for enhanced maternal and perinatal care in rural Nepal: opportunities and challenges. Ann Med Surg. 2024;86(11):6598–601. 10.1097/MS9.0000000000002588. PMC11543219 39525739

[62] Sanders JH, Bashshur RL. Challenges to the implementation of telemedicine. Telemed J Off J Am Telemed Assoc. 1995;1(2):115–23. .115 PubMed PMID: 10165330. 10.1089/tmj.1.1995.1.115 10165330

[63] Hinton L, Dakin FH, Kuberska K, Boydell N, Willars J, Draycott T, et al. Quality framework for remote antenatal care: qualitative study with women, healthcare professionals and system-level stakeholders. BMJ Qual Saf. 2022. 10.1136/bmjqs-2021-014329. bmjqs-2021-014329. PMC11041557 35552252

[64] Mahapatra P, Sahoo KC, Pati S. A longitudinal qualitative study on physician experience in managing multimorbidity across the COVID-19 pandemic in Odisha, India. Scientific Reports [Internet]. 2024. Available from: https://www.nature.com/articles/s41598-024-60473-0.pdf. 10.1038/s41598-024-60473-0 PMC11150371 38834635

[65] Aggarwal R, Guleria K, Sharma AK, Srivastava H, Aggarwal R. TELE-ANC model in public sector in India: a feasible option to sustain quality antenatal care. Int J Pregnancy Child Birth. 2023;9(5):136–43. 10.15406/ipcb.2023.09.00292.

[66] Venkataraman A, Fatma N, Edirippulige S, Ramamohan V. Facilitators and Barriers for Telemedicine Systems in India from Multiple Stakeholder Perspectives and Settings: A Systematic Review. Telemed E-Health. 2024;30(5):1341–56. 10.1089/tmj.2023.0297. 38206654

