# Supplementary material for: Telemedicine as a “digital umbilical” for maternal and reproductive healthcare: a qualitative study from health and wellness centers in rural West Bengal, India
Source: BMC Health Serv Res. 2026 Jul 6;26:1337. doi: 10.1186/s12913-026-14916-1 (PMC13628818; doi:10.1186/s12913-026-14916-1)
Supplement: Supplementary file 1 [file 12913_2026_14916_MOESM1_ESM.docx]

### Table S1. List of Participants in the study:

| **Participant No.** | **Designation** | **Area of Functioning** | **Age (Years)** | **Sex** |
| --- | --- | --- | --- | --- |
| 1 | Community Health Officer (CHO) | Health and Wellness Centre (HWC) | 31-35 | Female |
| 2 | Community Health Officer (CHO) | Health and Wellness Centre (HWC) | 36-40 | Female |
| 3 | Community Health Officer (CHO) | Health and Wellness Centre (HWC) | 26-30 | Female |
| 4 | Community Health Officer (CHO) | Health and Wellness Centre (HWC) | 31-35 | Female |
| 5 | Community Health Officer (CHO) | Health and Wellness Centre (HWC) | 26-30 | Female |
| 6 | Community Health Officer (CHO) | Health and Wellness Centre (HWC) | 26-30 | Female |
| 7 | Community Health Officer (CHO) | Health and Wellness Centre (HWC) | 36-40 | Female |
| 8 | Community Health Officer (CHO) | Health and Wellness Centre (HWC) | 36-40 | Female |
| 9 | Community Health Officer (CHO) | Health and Wellness Centre (HWC) | 46-50 | Female |
| 10 | Community Health Officer (CHO) | Health and Wellness Centre (HWC) | 36-40 | Female |
| 11 | Community Health Officer (CHO) | Health and Wellness Centre (HWC) | 26-30 | Female |
| 12 | Community Health Officer (CHO) | Health and Wellness Centre (HWC) | 26-30 | Female |
| 13 | Community Health Officer (CHO) | Health and Wellness Centre (HWC) | 31-35 | Female |
| 14 | Community Health Officer (CHO) | Health and Wellness Centre (HWC) | 26-30 | Female |
| 15 | Community Health Officer (CHO) | Health and Wellness Centre (HWC) | 41-45 | Male |
| 16 | Doctor (Medical Officer/MO) | Primary Health Centre (PHC) | 36-40 | Male |
| 17 | Doctor (Medical Officer/MO) | Primary Health Centre (PHC) | 31-35 | Male |
| 18 | Doctor (Medical Officer/MO) | Rural Hospital (RH) | 26-30 | Female |
| 19 | Doctor (Block Medical Officer of Health / BMOH) | Administration; Block Headquarters | 31-35 | Male |
| 20 | Doctor (Block Medical Officer of Health / BMOH) | Administration; Block Headquarters | 36-40 | Female |
| 21 | Doctor (District Nodal Officer: Telemedicine) | Administration; District Headquarter | 51-55 | Male |
| 22 | IT Staff | IT; District Headquarter | 26-30 | Female |
| 23 | IT Staff | IT; District Headquarter | 31-35 | Male |
