# Supplementary material for: Telemedicine as a “digital umbilical” for maternal and reproductive healthcare: a qualitative study from health and wellness centers in rural West Bengal, India
Source: BMC Health Serv Res. 2026 Jul 6;26:1337. doi: 10.1186/s12913-026-14916-1 (PMC13628818; doi:10.1186/s12913-026-14916-1)
Supplement: Supplementary file 2 [file 12913_2026_14916_MOESM2_ESM.docx]

### Table S2. Interview Guide

***Community Health Officers (CHO)/ Doctor/ Specialist/ Health Heads Interview Guidelines***

[Note: The following section is to be prefilled before starting the interview.]

| **Section 1:** Identifiers | | | |
| --- | --- | --- | --- |
|  |  |  |  |
| **CODE** |  | **Options / Response** | **Remark** |
| 101 | ID Number |  |  |
| 102 | District Name | 1- Howrah |  |
|  |  | 2- Nadia |  |
| 103 | Block Name: |  |  |
| 104 | CHC Name: |  |  |
| 105 | HWC/ Health Facility Name: |  |  |
| 106 | Health Facility Type: | 1- AB-HWC-SC |  |
|  |  | 2- AB-HWC-PHC |  |
|  |  | 3- CHC |  |
|  |  | 4- Others |  |
| 107 | Place of Interview: |  |  |
| 108 | Telemedicine Facility Available in the Health Facility: | 1-Yes |  |
|  |  | 2-No |  |

101	ID: Auto-generated according to fill up of the district code, interview number, block details, and health facility type

102	District Name: To be selected among the options

103	Block Name: To be filled in upper case

104	CHC Name: To be filled in upper case

105	HWC / Health facility Name: To be filled in upper case

106	Health facility type: Select among the options, if others please mention the health facility type in uppercase

107	Coordinates: To be fetched by the software

108	Telemedicine Facility Available in the **105: health facility Name**: If the telemedicine facility is available according to the line list, then yes else no

[ I will ask you some details about you to better understand your work in health service in **105: health facility** **name** facility]

| **Section 2: Health Worker Details** | | | |
| --- | --- | --- | --- |
|  |  |  |  |
| **CODE** |  | **Options / Response** | **Remark** |
| 201 | Sex | 1- Male |  |
|  |  | 2- Female |  |
| 202 | What is your current age in completed years? |  |  |
| 203 | What is your highest educational qualification? | 1- MD/ MS/ MPH/ Post MBBS Diploma or Higher |  |
|  |  | 2- M.Sc. Nursing or Higher |  |
|  |  | 3-MBBS |  |
|  |  | 4-AYUSH |  |
|  |  | 5- B.Sc. Nursing |  |
|  |  | 6- Diploma in Nursing |  |
|  |  | 7- Others |  |
| 204 | What is your current designation? | 1- Specialist Doctor |  |
|  |  | 2- Senior Medical Officer |  |
|  |  | 3- Medical Officer |  |
|  |  | 4- BMOH |  |
|  |  | 5- PHN |  |
|  |  | 6- CHO |  |
|  |  | 7- Others |  |
| 205 | How many years of total experience do you have in health services, excluding the internship? |  |  |
| 206 | How many years of experience do you have in your current job profile? |  |  |
|  |  |  |  |

201	Sex: To be selected among the options

202	Age: Write in absolute number

203	Education: Select the Highest attained degree/ certificate among the options if others, please mention them in the upper case

204	Designation: Select the designation among the options if others, please mention them in the upper case

205	Total Years of experience: Please put the absolute number in completed years

206	Years of experience in the current designation: Please put the absolute number in completed years

**Key Informant Interview Guidelines**

**Objective:** The basic objective of this tool is to get the information from the health workers about the telemedicine service, process, patients, facilitators and barriers while implementing the services etc.

**Purpose:** The purpose of key informant interviews is to collect information from the service providers perspective towards telemedicine services with their particular knowledge and understanding, these experts can provide insight on the nature of problems and recommend solutions.

**Key Informant Interview Checklist**

[ Now, to understand the details of the telemedicine service and the process I will ask you certain question related to these themes. Please answer those according to your understanding.]

I will appreciate if you can tell me about the nature of your work/role/responsibility

What are the different works you need to do regarding the telemedicine intervention?

**Telemedicine process**

How was telemedicine introduced to the health and wellness Centre (**Probe**: From the stating of the initiative)?

How is the selection of beneficiaries is done for the telemedicine consultation (**Probe**: Details registered before online consultation and selection)?

Can you please elaborate on the process of telemedicine consultation in the HWC (**Probe**: What are steps in a telemedicine consultation process)?

**Understanding the Telemedicine services**

Do you believe the telemedicine system is beneficial for the patients?

**Probe**: Please elaborate so I can understand more

How does it benefit patients?

In what ways did you find telemedicine is different from physical consultation?

What type of patients do you all encounter during the telemedicine consultation?

**Probe**: According to diseases, Age Groups. Educational background, Gender, Socio-economic background)

What are the problems patients face during the consultation process?

**Probe**: System, language, technical such as audio -visual, waiting time etc.

How the follow up process being done of a patient?

**Probe**: Patient track, Medicine dispenses, Tests, Re consultation

Interaction experience with other parties or doctors or specialists and beneficiary?

How data for the consultation is being collected?

**Prob**e: Type of data Collection, Storage, Reporting both offline and online

How the Supervision of the process is being done?

How Training of Staffs being undertaken for smooth service delivery?

**Barriers of Telemedicine**

Can you please tell us the initial constraints for implementing the initiative?

How would you manage to overcome those?

What are the challenges that are crucial to smoothening the process?

What are the main problems do you or health staffs like CHO, Doctors/ Specialist face consultation process or overall?

**Probe**: System, Equipment, Connectivity, Space, Timing, supervision

How can the telemedicine system be improved for better functioning, quality care provided, and access increased?

**Probe**: System, Process, Human Resource

What are the problems patients face during the consultation process?

How the monitoring process is done to identify whether the initiative is running smoothly?

**Facilitators**

What are the things helps to smoothly run the system? Please elaborate

How do we improve the telemedicine system for better functioning, provide quality care, and increase access?

What are the key elements that can help improve the services?

**Probe**: System, Process, Human Resource, Others

Sum up the points

Thanking the participants
