# Supplementary material for: Telemedicine as a “digital umbilical” for maternal and reproductive healthcare: a qualitative study from health and wellness centers in rural West Bengal, India
Source: BMC Health Serv Res. 2026 Jul 6;26:1337. doi: 10.1186/s12913-026-14916-1 (PMC13628818; doi:10.1186/s12913-026-14916-1)
Supplement: Supplementary file 3 [file 12913_2026_14916_MOESM3_ESM.docx]

Table S3.1. The Access vs. Quality Matrix in Hybrid Telemedicine Maternal Care

| Feature | The Access Benefit (Pros) | The Quality Deficit (Cons) |
| --- | --- | --- |
| **Geography** | Overcomes a 9-11km distance barrier. | **Waiting Time:** Patient saves travel time but loses time waiting for the connection (up to 2 hrs). |
| **Economics** | Zero cost; saves daily wages. | **Supply Chain:** Free consult is useless if free medicines are out of stock (forcing purchase). |
| **Clinical** | Access to a specialist (Gynecologist). | **Assessment:** The specialist cannot palpate/see; defaults to "Refer to OPD" without resolving the issue. |
| **Psychology** | "Big Doctor" builds confidence. | **Interaction:** Lack of video/eye-contact reduces rapport; patient speaks to a "black screen." |

### Table S3.2. Operational Friction Points for Hybrid Telemedicine model

| Component | Friction Description | Impact on Maternal Health |
| --- | --- | --- |
| **Targets** | CHO Target: 10 -15 cases/ day; MO Target: 75 cases/day. | Leads to **"Continue All"** prescriptions; dangerous for case management. |
| **Portal** | Version 2.0: Complex UI, AI questions. | Increases registration time to **15-20 mins**; reduces patient throughput. |
| **Network** | Internet instability | Forces **"Store and Forward"**; unsafe for acute pregnancy complaints. |
| **Hardware** | Printer/Laptop failure. | **Handwritten Prescriptions** lose the authority/legitimacy of the printed doctor's prescription. |
