# Supplementary material for: Telemedicine as a “digital umbilical” for maternal and reproductive healthcare: a qualitative study from health and wellness centers in rural West Bengal, India
Source: BMC Health Serv Res. 2026 Jul 6;26:1337. doi: 10.1186/s12913-026-14916-1 (PMC13628818; doi:10.1186/s12913-026-14916-1)
Supplement: Supplementary file 4 [file 12913_2026_14916_MOESM4_ESM.docx]

Table S4. Health Staff Sentiment Disparity: Access Success Versus Clinical Quality Failure in Hybrid Telemedicine

| Staff Category | Positive Sentiment (%) | Negative/ Critical Sentiment (%) | Neutral/ Mixed Utility (%) | Primary Focus Area (Maternal/ Reproductive Health Context) |
| --- | --- | --- | --- | --- |
| **Consulting Physicians (MOs)** | 18.8% | **50.0%** | 31.3% | Low professional confidence; high clinical risk perception due to lack of physical exam and reliance on inadequate CHO data. |
| **Frontline Providers (CHOs)** | 36.0% | **45.0%** | 18.0% | Systemic pressure (targets), long waits, and frustration over clinical communication failures (e.g., video denial, rushed consults). |
| **Administrative Doctors (BMOH/Nodal)** | **40.0%** | 24.0% | 36.0% | Policy success: high confidence in macro-level benefit (access, load reduction), viewing telemedicine as a strategically necessary support system. |
| **Technical Support (IT Staff)** | 30.0% | **50.0%** | 20.0% | Infrastructural reality: high critical rating driven by network instability, slow portal speed, and lack of dedicated manpower. |
